# Looking for a sepsis source

**DOI:** 10.1186/s13054-019-2715-z

**Published:** 2020-01-14

**Authors:** Damien Contou, Nicolas de Prost

**Affiliations:** 1 Service de Réanimation Polyvalente, Centre Hospitalier Victor Dupouy, 69, rue du Lieutenant-Colonel Prud’hon, 95100 Argenteuil, France; 2 Service de Réanimation Médicale, Hôpital Henri Mondor, Assistance Publique-Hôpitaux de Paris, 51 Avenue du Maréchal de Lattre de Tassigny, 94000 Créteil, France

Dear Editor,

We read with great interest the editorial written by de Waele and Sakr [1], in which the authors described their pragmatic strategy on how to search the source of a sepsis. It appears of paramount importance to precise that searching for a source of infection does not always mean finding a source of infection. Indeed, the lack of documentation (clinical or microbiological) of a source of infection during the 24 first hours of a septic shock is a common but disturbing and challenging clinical scenario reflected by the classical question “what does my patient have?” often heard during the morning rounds in many ICUs.

We recently reported in the Journal the results of a pragmatic multicenter prospective observational cohort study [2] including 508 patients admitted to the ICU for a suspicion of septic shock. It is worth notifying that more than a quarter of them (*n* = 134/506, 26%) had no source of infection nor microbiological documentation retrieved 24 h after shock onset (defined as the start of vasopressor infusion), despite an exhaustive diagnostic work-up. Indeed, these patients underwent more diagnostic testing with more imaging procedures—including computed tomography of the chest and abdomen and echocardiography—during the first 24 h of shock management, as compared to those with a source of infection identified within the first 24 h of shock. These patients without an “early confirmed septic shock” eventually had either a source of infection or a microbiological documentation retrieved after the 24 first hours (*n* = 37/134, 28%)—mostly a respiratory, urinary, or abdominal sepsis—or a sepsis mimicker (*n* = 59/134, 44%)—mostly an adverse event of drugs, an acute mesenteric ischemia, or a malignancy—or a shock of unknown origin (*n* = 38/134, 28%). Mortality did not differ between patients with an early confirmed septic shock and those with a non early confirmed septic shock.

Intensivists should be aware that the absence of a source of infection is not so uncommon in the first 24 h of management of a patient with a suspected septic shock. A source of infection may be diagnosed later, but the hypothesis of a sepsis mimicker should be suspected in such a context.

## Authors, response

Jan J. De Waele and Yasser Sakr

To the editor,

We agree with Drs. Contou and de Prost that in some patients with sepsis or septic shock, an infection diagnosis cannot be established in the first 24 h. In their study on patients admitted to 10 ICUs in France, indeed a septic shock diagnosis could not be confirmed in 26% of patients [2]. In the majority of patients without confirmed septic shock, either no cause or another cause of the shock could be established, and only in less than 1 out of 3 patients, an infection cause was established later—most of these were pneumonia and urinary tract or abdominal infections. However, when only considering patients with a final diagnosis of septic shock, this diagnosis was in fact confirmed within 24 h in over 90% of the patients (374/411). The message here is that when an infection source cannot be identified within a 24-h timeframe, it is more likely that there is an alternative explanation for the shock and no infection is present. This does not mean however that the search for the infection source should not be continued. These data align with nicely the finding by Klein Klouwenberg et al. who demonstrated that in 1 out 6 patients in whom sepsis or septic shock was suspected in the emergency department, eventually no infection was documented [3].

Clearly this demonstrates that we remain poor at diagnosing sepsis—read diagnosing infection—and that we should acknowledge that in many patients in whom we suspect infection, in fact there is none. However, using a systematic approach, we should try to maximize the chances of establishing a final diagnosis of septic shock [1].

## Data Availability

Not available.
